# Non-Lethal Sampling Supports Integrative Movement Research in Freshwater Fish

**DOI:** 10.3389/fgene.2022.795355

**Published:** 2022-04-25

**Authors:** Matt J. Thorstensen, Carolyn A. Vandervelde, William S. Bugg, Sonya Michaleski, Linh Vo, Theresa E. Mackey, Michael J. Lawrence, Ken M. Jeffries

**Affiliations:** Department of Biological Sciences, University of Manitoba, Winnipeg, MB, Canada

**Keywords:** sublethal, aquatic, genomic, RNA-seq, transcriptomic, molecular, biopsy, interdisciplinary

## Abstract

Freshwater ecosystems and fishes are enormous resources for human uses and biodiversity worldwide. However, anthropogenic climate change and factors such as dams and environmental contaminants threaten these freshwater systems. One way that researchers can address conservation issues in freshwater fishes is *via* integrative non-lethal movement research. We review different methods for studying movement, such as with acoustic telemetry. Methods for connecting movement and physiology are then reviewed, by using non-lethal tissue biopsies to assay environmental contaminants, isotope composition, protein metabolism, and gene expression. Methods for connecting movement and genetics are reviewed as well, such as by using population genetics or quantitative genetics and genome-wide association studies. We present further considerations for collecting molecular data, the ethical foundations of non-lethal sampling, integrative approaches to research, and management decisions. Ultimately, we argue that non-lethal sampling is effective for conducting integrative, movement-oriented research in freshwater fishes. This research has the potential for addressing critical issues in freshwater systems in the future.

## Introduction

Communities around the world rely on freshwater ecosystems for natural resources, including impoverished groups that rely on freshwater resources for survival (Sultana et al., 2003; Millenium Ecosystem Assessment Board, 2005; Vo, 2016). Fresh water is used directly by humans in numerous ways, including drinking, farming, and transportation, but human demand for fresh water is increasing because of population growth and overexploitation (Strayer and Dudgeon, 2010; Dudgeon, 2019). Freshwater fishes are critical, and often overlooked, sources of food security for hundreds of millions of people globally, providing otherwise unavailable protein *via* freshwater fisheries (McIntyre et al., 2016; Dudgeon, 2019), in addition to essential nutrients for human diets such as omega-3 and omega-6 fatty acids (Steffens and Wirth, 2005). Given the importance of freshwater ecosystems to biological diversity and human resource needs, conservation issues in fresh water are paramount (Dudgeon, 2019; Su et al., 2021). Aquatic species are increasingly imperiled as human needs increase, an issue exacerbated by the tendency for the tragedy of the commons to be present among communities that use freshwater resources (Strayer and Dudgeon, 2010; Dudgeon, 2019). Human activities have changed natural habitats in ways that include overexploitation, physical alterations of natural structures and ecosystem components (e.g., dams, non-native species), as well as water pollution as a result of industrial development (Strayer, 2008; Dudgeon, 2019). Anthropogenic climate change has broad, insidious effects on freshwater ecosystems that include rising temperatures, changing flow patterns, and more frequent extreme events that are all already apparent throughout many freshwater systems (Dudgeon, 2019). Conservation action is therefore necessary in many freshwater systems. Integrated terrestrial-freshwater conservation planning can increase freshwater species protection by 600% with marginal drawbacks, demonstrating the effectiveness of conservation action in freshwater systems (Leal et al., 2020).

Freshwater ecosystems contribute around 40% of global fish diversity, 25% of global vertebrate diversity, and ∼10% of all known species despite covering <1% of the earth’s surface (Dudgeon et al., 2006; Strayer and Dudgeon, 2010; Reid A. J. et al., 2019; Radinger et al., 2019; Leal et al., 2020). This diversity is reflected in species richness, phylogenetic diversity, and functional diversity, which are disproportionately high for freshwater ecosystems (Dudgeon, 2019; Su et al., 2021). For example, the Mekong, Congo, and Amazon Rivers are some of the most fish-diverse systems globally with thousands of fishes in each, and are characterized by their relative abundances of fresh water (Sverdrup-Jensen, 2002). The great diversity of life present in fresh water contributes to valuable ecosystem services, but also underscores the importance of the conservation issues that freshwater ecosystems face (Dudgeon, 2019). Physical structures such as dams can impede movement for freshwater organisms, an issue exemplified by the observation that only 37% of rivers longer than 1,000 km remain free-flowing (Dudgeon, 2019; Grill et al., 2019). Responses to global climate change often require compensatory movements by freshwater organisms, such as in finding cool water (Dudgeon, 2019). These responses are stymied by impediments to movement in freshwater systems. One reason that movement is especially important in freshwater is that these habitats are often interspersed among terrestrial habitats, in a matrix characterized by varying levels of connectivity (Griffiths, 2015; Mushet et al., 2019).

Research on freshwater fishes therefore has the potential to address critical issues in human resource needs and conservation of biodiverse ecosystems. One potential avenue of research is the study of fish movement. Movement in general is a fundamental trait for many organisms, underlying survival, reproduction, and population persistence (Nathan et al., 2008). Moreover, movement with respect to individuals is foundational for considering other mechanistic components of movement, such as the internal state of the individual or the effects of the external environment (Nathan et al., 2008). Because non-lethal sampling provides an opportunity for physiological or genetic samples to be Because non-lethal sampling provides an opportunity for physiological or genetic samples to be taken from individuals whose movement may be tracked (Prince et al., 2017; Jeffries et al., 2021), non-lethal sampling is consistent with this framework of individual-focused movement as it can provide a snapshot of the internal state of the individual at the time of sampling.

The objective of this review is to support integrative research in freshwater fishes by presenting an overview of methods for studying movement in conjunction with the physiological and genetic approaches that have been linked with movement. Beyond specific methods, various factors that we consider important to this integrative movement research are discussed. This review is not intended to provide an exhaustive survey of the relevant literature. Instead, we highlight possible approaches for linking movement with other observations, broadly classified into physiology and genetics. First, *Methods for Studying Movement* in the context of tagging methods and movement data analyses are addressed as a foundational step for practicing research on fish movement. In *Methods for Connecting Movement and Physiology*, different examples of whole-organism and molecular physiology approaches to studying movement are provided as an overview of possible research avenues. In *Methods for Connecting Movement and Genetics*, population genetics, quantitative genetics, and association tests are presented in the context of movement research. In *Further Considerations*, fish handling, collecting molecular data, the ethics of non-lethal sampling, integrating data, and applications of movement research to management are presented as ways that movement-focused research may be more effectively practiced and used. For each type of sampling method, we discuss how the sample is collected, the types of movement-relevant information it can provide, and potentially ways the sample may be used to support conservation or management decisions with case studies from the literature. While this review is focused on freshwater fish movement and non-lethal sampling, several marine fish and also non-fish studies are discussed because they illustrate concepts relevant to freshwater fishes.

## Methods for Studying Movement

### Tagging Methods

Animal movement can be tracked with several methods (Table 1). These methods generally fall under two approaches: mark-recapture and telemetry. In mark-recapture, organisms are observed over multiple sampling events where small tags (e.g., anchor, visible implant elastomer (VIE), coded wire, or electronic tags) are attached to fish and used to individually identify these organisms at a tagging event and subsequent recapture (Hale and Gray, 1998; Walsh and Winkelman, 2004; Hohn and Petrie-Hanson, 2013; Thorstad et al., 2013; Cayuela et al., 2018). The mark-recapture approach is useful for estimating dispersals, but it also allows the modeling of population size through rates of recapture (Nichols, 1992; Cayuela et al., 2018). A related approach is a VIE tag, where coloured tags are injected into fish in externally visible places (Walsh and Winkelman, 2004; Hohn and Petrie-Hanson, 2013). In contrast to mark-recapture, telemetry offers a different approach for tracking movement. Both radio and acoustic telemetry arrays can be built for autonomous operation, enabling the tracking of multiple tagged individuals without a researcher needing to track each individually (reviewed in Cooke et al., 2008; Donaldson et al., 2014). As multiple fish species may be tagged across fresh water and salt water (Hussey et al., 2015), acoustic telemetry arrays have the potential for increased tracking of a variety of fish species and studying interactions among entire aquatic communities (Hussey et al., 2015; Enders et al., 2019; Rudolfsen et al., 2021). Acoustic tags sensitive to predation events are used to track predators as well, for a mean of 66.5 h or 75.6 h after predation depending on the model of tag used and ambient temperatures (Halfyard et al., 2017; Schultz et al., 2017). In one test of the technology, predation transmitters accurately verified a predation event 90% of the time, although mean time from feeding event to trigger time of the tag was 59.2 h (Schultz et al., 2017).

**TABLE 1:** Methods for studying movement in freshwater fishes. Methods are broadly classified into mark-recapture and telemetry-based approaches. Technology provides the commonly used name for each method and description provides a brief summary of how the method operates. Advantages, disadvantages, and representative literature for each method are also provided.

	Technology	Description	Advantages	Disadvantages	Literature
**Mark-recapture**	*Anchor tags*	Attached to organisms as an identification tool at a tagging event and subsequent recapture	Low cost, well-established method	Limited movement information value, from only mark and recapture events	Thorstad et al. (2013)
			Can be used to estimate population size		
	*Passive integrated transponder tags (PIT)*	Small electronic tags, often internally implanted in an organism. Researchers detect the PIT tag with a handheld device or automated station	Weigh < 1 g	Short detection distance of the readers (<1 m)	Thorstad et al. (2013)
			No battery is needed		Hedger et al. (2013)
			Lifetime of several decades		
	*Visible Implant Elastomer Tags (VIE)*	Colored tags where elastomer is injected as a liquid and solidifies. Tags are placed beneath clear or translucent tissue and remain externally visible	Useful in a wide variety of species	Limited utility when many individuals must be tracked simultaneously	Hohn and Petrie-Hanson (2013)
			Can be used in very small organisms		
			Flexible		Walsh and Winkelman, (2004)
			Biocompatible		
	*Close-kin mark-recapture*	Employed to track individuals and populations based on genotype-derived estimates of kinship. The genetic information is collected *via* adipose fin clips	Only fin clip is required from fish	Molecular resources required	Bravington et al. (2016)
					Marcy-Quay et al. (2020)
**Telemetry**	*Pop-up satellite tag (PSAT)*	Archival tag that collects information for a specific period and relays that information to receivers *via* satellites	Enables large scale ocean migration studies of large fish	Tags are large and must be attached externally	Thorstad et al. (2013)
			Do not need to be recovered to retrieve data		Raby et al. (2017)
			Encourages public participation as a receiver array is not necessary and the public can report the captured tags		
	*Global positioning system tag (GPS)*	Tags that gather accurate spatial information from Global Positioning System satellites and can transmit or store that information	Enables tracking of very small individuals due to radio and GPS tag miniaturization	Limited in aquatic systems because signals must be transmitted at or near the water’s surface	Sims et al. (2009)
			Very accurate	Often requires a tether to follow the fish, allowing the tag to get GPS data from near or at the water’s surface	Evans et al. (2011)
					Riding et al. (2009)
	*Acoustic & Radio Arrays*	A receiver array is established, then fish are tagged with acoustic or radio transmitters. Data is collected from receivers, revealing fish locations over time	Acoustic arrays work in both fresh water and saltwater. Radio arrays work in saltwater	High initial cost for establishing array	Hussey et al. (2015)
			Autonomous operation	Precision of movement information limited by the density of the receiver array	Cooke et al. (2008)
			Cost-efficient after array is established		Donaldson et al. (2014)
			Multiple species can be monitored at once	Movement information limited to the spatial coverage of the array	Enders et al. (2019)
	*Predation transmitter (acoustic tag)*	Acoustic tags that are sensitive to predation events. Transmitters change their acoustic identifier in response to acidic conditions in a predator’s stomach. This shift in identifier marks a predation event and tags can record the predator’s movement	Accurate (shown to verify a predation event 90% of the time)	Limited data collection time after predation event	Halfyard et al. (2017)
					Schultz et al. (2017)
			Could aid in describing aspects of predator feeding behavior and physiology		Weinz et al. (2020)
					Klinard et al. (2019)

As capture, handling, and surgery can be acutely stressful for the animal, such procedures may induce an acute stress response resulting in physiological and behavioural perturbations (Wendelaar Bonga, 1997). This is an important consideration as tagging stress may contribute to higher rates of post-release predation/mortality and altered behavioural patterns (Mäkinen et al., 2000; Danylchuk et al., 2007; Le Pichon et al., 2015; Wilson et al., 2017), which would be deleterious to the project’s objectives. For example, Atlantic salmon (*Salmo salar*) caught through gill netting (i.e., slower time from capture to release, high potential for injury and mucus loss) or rod and reel angling (i.e., faster time from capture to release) exhibited distinct differences in post-release movement patterns, suggesting that capture methods do have a role in affecting acute behaviours (Mäkinen et al., 2000). To ensure that post-release behaviour/movement is marginally impacted, efforts should be made to ensure that capture and handling is minimized to reduce stress-related effects. The specific protocols used will be project-, species-, and context-dependent but should include measures that either limit handling/collection times or reduce injury to the fish. For example, rod and reel angling is used in a number of settings to collect animals for tagging (Heupel and Hueter, 2001; Gillis et al., 2010) as it has short capture durations, generally has a minor physiological impact on the fish (Brownscombe et al., 2017), and can allow for more targeted collection (c.f. gill nets or long lines). Similarly, using electro anaesthesia over chemical anaesthetics for tagging manipulations can also help to hasten tagging procedures owing to electro anaesthesia’s shorter induction and recovery times (Reid et al., 2019). In addition to reducing holding times, the tagging environment is also important in minimizing stress and injury to the animal. The fish should be held at its environmental temperature with its gills submerged to facilitate normal gas and ion exchanges. This can be achieved using a padded, water filled trough with a continual flow of fresh water, which promotes ease of access during the tagging procedure and less stress for the fish than handling without water flow (Lawrence et al., 2020). Censoring data in the days or weeks following tagging can minimize the effects of tagging stress represented in subsequent analyses of movement, as well (Wilson et al., 2017). Together, the use of proper collection methods, minimal handling, expediting tagging, and data handling can help to ensure that stress-related effects on post-release behaviours are minimized.

### Movement Analyses

Movement data connects the studies in this review and its careful analysis is of fundamental importance for any project that seeks to connect movement with other non-lethally sampled variables. The field of movement ecology has developed sophisticated methods for analyzing such data. Previously, random walks with Hidden Markov Models were a common approach but more recently, connections between complex movement behaviours and landscape or geographical features have become more widely used (Schick et al., 2008; Seidel et al., 2018). Specialized statistical approaches have been developed, such as in assessing spatial autocorrelation and in specific classes of analysis techniques (reviewed in Long and Nelson, 2013). For example, just in the statistical computing environment R, 58 packages were identified as contributing to movement analyses, 11 of which had good or excellent documentation (Joo et al., 2020; R Core Team, 2021). The R package actel has been developed to reproducibly analyze acoustic telemetry array data, an increasingly common source of movement information in fishes (Flávio and Baktoft, 2021). Meanwhile, Bayesian state space models have been used to infer movement paths with telemetry data, showing a possible direction for movement analyses in the future (Munaweera Arachchilage et al., 2021). Regardless of the approach to movement analyses undertaken by researchers, careful interpretation of movement data is necessary for subsequent integrative analyses.

## Methods for Connecting Movement and Physiology

### Physiology and Movement

The biological impact of stressors associated with movement can be assessed through non-lethal sampling for transcriptomic (mRNA) and biochemical analyses (e.g., enzymes, hormones, osmolytes), as well as additional physiological data to investigate their roles at both the individual and population-level scales (Metcalfe et al., 2012; Connon et al., 2018; Jeffries et al., 2021). For example, salmonid (*Oncorhynchus* spp. *& Salmo salar*) migration has been studied in depth from physiological perspectives of thermal stress, cardiorespiratory performance, disease states, and stress responses using a combination of telemetry, physiological, and genomic methods (Cooke et al., 2008, 2012; Stich et al., 2015). Increased baseline levels of a commonly studied hormone, cortisol, has been linked to sooner river exit times but a lower likelihood of reaching the sea in Atlantic salmon and sea trout (*S. trutta*), with implications for a tradeoff between stress and migration readiness (Birnie-Gauvin et al., 2019). Elevation of cortisol and its decline during migration was consistent between landlocked kokanee salmon (*Oncorhynchus nerka kennerlyi*) and anadromous sockeye salmon (*O. nerka nerka*) sampled before, during, and after migration, providing evidence that cortisol is part of an endogenous system that is associated with migration and insights into the timing of movements in these species (Carruth et al., 2000). Physiological metrics have also been used to study Atlantic salmon, with investigation of smolt survival through telemetry and physiological assays demonstrating that individuals with the highest levels of gill Na+, K+-ATPase activity incurred 25% lower mortality than those with the lowest Na+, K+-ATPase activity, linking physiological preparedness for migration with survival success (Stich et al., 2015).

Similar to patterns in more southerly salmonids, arduous migratory journeys exacerbated by changing climates and stressors can influence selective pressures and alter the physiology of subsequent generations as observed in Arctic char (*Salveliunus alpinus*) in northern Canada. Using a combination of non-lethal sampling for genomics and telemetry, Moore et al. (2017) demonstrated increased selection pressures (*via* outlier markers) on cardiac genes of char as migratory length and elevation gain increased. These genes were related to skeletal muscle differentiation and heart development, actin binding in the sarcomere Z-disc, glycogen breakdown into glucose, and the conversion of glycerol to glucose (ibid). In a related study, physiological metrics of increased thermal maxima, increased aerobic capacity, and decreased heart mass were also associated with increased population migratory difficulty (Gilbert, 2020). Together these studies apply a variety of techniques linking genetics, physiological capacity, and telemetry to investigate the migratory challenges faced by different Arctic char populations. Their endurance capabilities for reproductive migration were assayed using a combination of non-lethal sampling, movement data, and classical physiological metrics.

Anthropogenic influences of aquaculture-related pathogens, contaminants, and water usage can alter the movement of fishes (Brander, 2007; Nathan et al., 2008; Bozinovic et al., 2011; Miller et al., 2014). Salmonids making reproductive migrations may be further challenged by increased, or novel, pathogen spillover from aquaculture facilities resulting in elevated infection pressure in a critical stage of their life cycle (Cicco et al., 2018; Mordecai et al., 2021). Additionally, hormones such as 17β-estradiol can be introduced into aquatic systems from anthropogenic activities and have been demonstrated to alter migration behavior in both Atlantic and sockeye salmon (Madsen et al., 2004; Veldhoen et al., 2013a). Infectious pathogen presence, populations, and abundance have been assessed using non-lethal sampling, using transcriptomic approaches paired with telemetry, to evaluate increasing predation and limited reproductive success in stressed and infected salmonids throughout their migrations (Jeffries et al., 2014; Furey et al., 2021; Vollset et al., 2021).

In freshwater systems, anthropogenic water allocation can impact the movement and impose restrictions to swimming passage, limiting the successful progression of fishes throughout their lifecycle. Dams and other diversions can lead to habitat fragmentation or alter flow states, decreasing hydrological connectivity and restricting fishes from habitat critically important for their survival and reproduction (Yoshiyama et al., 1998; Jeffres et al., 2006; McKay et al., 2013; O’Hanley et al., 2013). In sockeye salmon monitored while crossing dam passages, using a combination of telemetry and biomonitoring, increased oxygen consumption (MO_2_) and anaerobic glycolysis while ascending the fishway indicated higher probability of mortality, while lower plasma glucose, earlier migration, and longer lake residency time upstream of the dam increased the probability that migrating fish reached their spawning sites (Roscoe et al., 2011; Burnett et al., 2014, 2017; Bett et al., 2020). For species like sturgeons, with poor swimming capabilities and lower success passing through dams, spillways, and diversions, these passages and the restrictions that they impose can lead to direct mortality or increased physiological stress leaving them vulnerable to predation as juveniles (Peake et al., 1997; Cheong et al., 2006; Cocherell et al., 2011; Poletto et al., 2014; Katopodis et al., 2019). By pairing movement data with non-lethal sampling and physiological metrics successfully, researchers can clarify the processes influencing, allowing, or promoting specific movement trajectories in response to anthropogenic water allocation.

### Tissue Biopsies

Tissue biopsies are an effective means to collect physiological, molecular, and environmental data for use in spatial movement projects. They can non-lethally provide insight into how physiological and environmental characteristics influence fish behaviour, spatial use, and ecological interactions, thereby linking multiple levels of biological scale (Cooke et al., 2008; Donaldson et al., 2014). As well, given their minimally invasive nature, the use of non-lethal biopsies is useful in settings where lethal samples are not obtainable, as in the case of species at risk, or where obtaining unadulterated behavioural/movement-based data is the key aim of the project. The non-lethal nature of such samples also means that large sample sizes can be readily obtained without substantially diminishing a studied population (Henderson et al., 2016). Non-lethal tissue biopsies in contemporary ecological studies typically sample tissues that are easily accessible and minimize stress on the animal.

**FIGURE 1:**
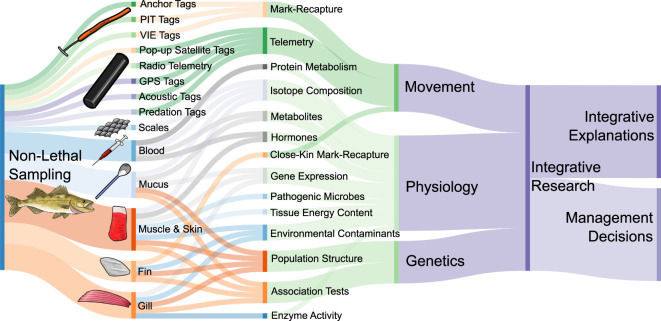
Conceptual diagram of different tagging and tissue sampling methods used for non-lethal sampling and movement studies in fishes. Specific sampling and tagging methods are linked to overall approaches in research, although for simplicity, only approaches presented in this review with examples are shown here. The different research approaches are categorized into movement, physiology, and genetics, which can be incorporated into integrative research projects. These integrative projects are useful for making informed management decisions and finding integrative explanations of natural phenomena. Therefore, movement, physiology, and genetics can all inform integrative explanations and management decisions *via* integrative research. Colour choices are arbitrary, and only for distinguishing elements of the diagram. Created with SankeyMATIC.

One of the most widespread biopsy approaches in spatial ecology involves the use of blood samples (Figure 1; Caputo et al., 2009; O’Toole et al., 2012; Taylor and Litvak, 2017; Thorstensen et al., 2021). While specific details on sampling methods can be found elsewhere (Houston, 1990; Lawrence et al., 2020), this approach is advantageous as it is quick, minimally invasive, and can provide insight into the fish’s physiological state (reviewed in Lawrence et al., 2020). The physiological metrics obtained from blood samples can be used to inform the proximate mechanisms driving fish movement and behavioural dynamics. For example, the use of blood sampling has been an integral component in understanding fisheries interactions and spawning migration of Pacific salmonids showing that indices of stress often correspond with poor migration success (Cooke et al., 2006, 2008; Drenner et al., 2018; Bass et al., 2019). In a recreational fishing context, blood sampling has been coupled with telemetry and monitoring to characterize the factors contributing to post-release survivorship and behaviour (Arlinghaus et al., 2009; Heberer et al., 2010; Tracey et al., 2016). Blood samples can also be used to indirectly characterize foraging and habitat use patterns through isotopic analyses when paired with telemetry datasets (Matich and Heithaus, 2014; Shipley et al., 2021). Blood sampling on live fish often involves caudal puncture where a small needle/syringe is inserted through the ventral surface of the caudal peduncle, piercing the dorsal vasculature (Lawrence et al., 2020). This method applies to a wide range of species, sizes, and settings, with smaller sized fishes requiring a slightly modified approach (e.g., lateral blood sampling in zebrafish; Zang et al., 2013, 2015). The sampling should occur in an environment that minimizes stress on the animal, restrains the fish adequately, and enhances ease of blood sampling. This is often achieved using a padded V-trough that the fish is held ventral side up to facilitate sampling access. The gills of fish can be artificially irrigated with water, or the trough filled with water to allow oxygen exchange (see Lawrence et al., 2020). As this method is minimally invasive, mortality rates are generally considered to be low making it an effective technique for non-lethal sampling (Lawrence et al., 2020).

Another common non-lethal method in fish biology includes the use of gill biopsies, which involves the removal of a small portion of the distal end of several gill filaments (McCormick, 1993). This approach is a relatively non-invasive means of tissue sampling and appears to have limited consequence on the fish’s post-release survival or growth/condition indices when compared to handling controls (McCormick, 1993; Martinelli-Liedtke et al., 1999; Cooke et al., 2005; Jeffries et al., 2014). However, some work has demonstrated that biopsied fish had higher post-release mortality rates, relative to non-biopsied conspecifics (Bass et al., 2020). As with blood sampling, a large breadth of information can be obtained from gill biopsies that can be informative in movement-based projects. Gill biopsies are being used extensively to quantify pathogenic microbes and host biomarkers of disease in Pacific salmonids (Miller et al., 2014; Chapman et al., 2021). Using molecular toolsets (e.g., qPCR, RNA-seq), gene expression patterns related to disease in the gill have been coupled with telemetry datasets, with links between disease burdens and migration activities (Jeffries et al., 2014; Teffer and Miller, 2019; Chapman et al., 2020; Stevenson et al., 2020; reviewed in; Chapman et al., 2021). Because the gill is one of the most important structures in regulating ion and acid base balance, waste excretion, and oxygen uptake (Evans et al., 2005), gill biopsies can also be used to derive important physiological responses to environmental challenges. This often includes quantifying activities of relevant enzymes/transporters (e.g., Na^+^/K^+^ ATPase; McCormick, 1993; Zhao et al., 2014; Gesto et al., 2015) or by assaying gene expression patterns (Rees et al., 2005; Norman et al., 2014; Guo et al., 2018). Consequently, gill biopsies can be useful in assaying responses to a wide array of treatments and environmental challenges (reviewed in Jeffries et al., 2021). In spatial ecology specifically, gene expression analyses can be combined with telemetry data to provide a more comprehensive assessment of how fine scale transcriptomic changes can influence fish movement patterns and behaviour (Madsen et al., 2004; Crossin et al., 2009; Evans et al., 2011). In some cases, gill biopsies have been proposed as an alternative to blood sampling as a means of collecting physiological metrics such as cortisol (Gesto et al., 2015). Similar to blood biopsies, non-lethal gill biopsies may not be feasible in smaller fishes, which might require that the animal be sacrificed to obtain a sample (ibid). However, this approach has been successfully used to take a non-lethal gill biopsy from sockeye salmon smolts (Jeffries et al., 2014).

Muscle, skin, and scale biopsies could also play an important role in movement-based studies (Figure 1). These techniques employ a similar approach, where a small tissue sample is removed using either a medical-grade biopsy punch device (e.g., Crawford et al., 1977; Tyus et al., 1999; Smith et al., 2015; Henderson et al., 2016; Bøe et al., 2020) or, in the case of skin from fin samples specifically, simply clipping a portion of the fish’s fin off (Sanderson et al., 2009; Galván et al., 2015; Wang et al., 2017). Scale samples may be removed from the exterior of the fish (Cunjak et al., 2005; TinHan et al., 2018). Both skin and muscle biopsies are minimally invasive and appear to have little impact on the fish’s health and post-release mortality with wounds healing within weeks in some instances (Crawford et al., 1977; Tyus et al., 1999; Pratt and Fox, 2002; Henderson et al., 2016; Bøe et al., 2020).

Non-lethal skin and muscle samples can provide a variety of metrics that are useful in spatial ecology projects. Historically, tissues derived from muscle, scale, and skin biopsies have been used in the quantification of accumulated environmental toxicants (Aerts et al., 2015; Alves et al., 2016; Gelsleichter et al., 2020; Charette et al., 2021; Stahl et al., 2021), isotopic compositions (Cunjak et al., 2005; Kim et al., 2012; Busst et al., 2015; Peterson et al., 2017; TinHan et al., 2018), pathogenic microbes (Bowers et al., 2008; Elliott et al., 2015), DNA (John Nelson et al., 2003; Dominik et al., 2010; Smith et al., 2018; Thorstensen et al., 2019), and physiological metrics such as hormonal levels and tissue energy contents (Fagan et al., 2012; Olsen et al., 2013; Verkamp et al., 2021). Koi (*Cyprinus carpio*) mucus was assayed for 11-ketotestosterone (Schultz et al., 2005), while mucus was also useful for stable isotopes analysis of rainbow trout diet (*Salmo gairdneri*) (Church et al., 2009), and DNA in Nile tilapia (*Oreochromis niloticus*) (Taslima et al., 2016). While these tissues can provide a great deal of insight, it is important to ensure that values derived from such samples are interpreted within the right context. For example, when measuring levels of mercury in skin and muscle biopsies in freshwater teleosts, the degree to which these values represent whole muscle mercury concentrations can vary (Baker et al., 2004; Piraino and Taylor, 2013; Stahl et al., 2021), highlighting the need for preliminary analysis and calibration. To date though, the use of non-lethal skin and muscle biopsies in movement-based studies appears limited, but biopsies provide a wealth of context-dependent biological information. Some potential links between biopsies and movement are presented in the following sections.

### Environmental Contaminants

Evaluating the effects of contaminants in fish often involves the removal of liver and/or muscle tissues during lethal sampling to examine chemical or biological indicators of toxicity (reviewed in Gray et al., 2002). These lethal samples can limit the quantity of samples one is able to collect, experimental design options, target species (i.e. species at risk), repeatability, and long-term analysis of individuals (Gray et al., 2002; Sanderson et al., 2009; Rodríguez-Jorquera et al., 2019; Jeffries et al., 2021). Because of these limitations, using non-lethal and minimally-invasive methods has become more common practice (Jeffries et al., 2021). Molecular responses to a range of contaminant exposure have been tested with many non-lethal samples, including muscle (Veldhoen et al., 2010; Somerville et al., 2020), fins (Andreasen et al., 2006; Veldhoen et al., 2013b, 2014; Baillon et al., 2016), mucus (Arukwe and Røe, 2008; Bahamonde et al., 2019; Bulloch et al., 2020), blood and plasma (Arukwe and Røe, 2008; Rodríguez-Jorquera et al., 2019), gill (Rees et al., 2005; Barriga-Vallejo et al., 2017), and scales (Aerts et al., 2015; Alves et al., 2016) (Figure 1). However, when it comes to the intersection of contaminants, non-lethal sampling, and molecular work, there are few examples that analyze aspects of movement. Contaminants can affect anadromous migrations, along with temperature changes, pathogens, and a multitude of other stressors, and therefore alter reproductive success and population survival (Veldhoen et al., 2013a). For example, Madsen et al. (2004) exposed Atlantic salmon to 17β-estradiol and 4-nonylphenol to evaluate enzyme activity, smolting, and migration success. Using non-lethal gill samples at multiple time points, Na^+^,K^+^-ATPase mRNA levels were analysed and found to be depressed in the 17β-estradiol and 4-nonylphenol treatments during smolting and migration. They also found that 17β-estradiol and 4-nonylphenol delayed smolting and migration, and increased mortality during migration compared to control fish. Exposure to contaminants has also been found to heighten sensitivity to external stressors, which may impact the long-term sustainability of migrating salmon populations (Lerner et al., 2007).

Locations of high residency time (e.g., spawning areas, areas with high food availability, migration routes) can also affect the contaminant burdens individuals face (Hoffman et al., 2020). For example, Heltsley et al. (2005) used mobile passive sampling devices (PSD) attached to tags on flathead catfish (*Pylodictis olivaris*) to determine location-specific exposure to polychlorinated biphenyls (PCBs) and organochlorine pesticides. To measure these concentrations, they evaluated if adipose fins could be used in replacement of lethal muscle samples. They found that the concentrations of PCBs and organochlorine pesticides in adipose fin samples correlated to lethal muscle tissue samples, providing an appropriate minimally invasive substitute for this species. They also found contaminate concentrations in the mobile PSDs correlate to the concentrations found in the stationary PSDs in several locations with varying PCB and organochlorine pesticides concentrations. The concept that the movement ecology of a species can affect their contaminant burden is significant, particularly in the context of point source pollution. Reduced movement or increased use of habitat at a point source of pollution may contribute to increased contaminant burden. By contrast, more transient movements through such an area are less likely to influence individuals’ contaminant burdens. These connections between contaminant burden and movement may have long term impacts on fish populations that can be monitored through physiological metrics.

### Isotopes

Stable isotope levels derived from non-lethally collected fin clips, scales, and skin have also been used to inform habitat use and foraging patterns in fishes when coupled with movement data (Cunjak et al., 2005; TinHan et al., 2018; Eggenberger et al., 2019; Aschettino et al., 2020). For example, stable isotopes in conjunction with PIT tags were used to distinguish between movement for foraging or to seek cool water refugia in Atlantic salmon (Cunjak et al., 2005). Here, *δ* ^13^C measured from muscle tissue was associated with downstream sites along the river system (Gray et al., 2004; Cunjak et al., 2005). Acoustic telemetry was paired with isotope analyses to determine that common snook (*Centropomus undecimalis*) of higher trophic levels moved more often into different zones than snook of lower trophic levels (Eggenberger et al., 2019). Similarly, research in seatrout (*Cynoscion nebulosus*), using acoustic telemetry paired with isotopic signatures from scales, revealed that fish resident to a recently restored oyster reef were larger and had narrower isotopic niches than their smaller, less reef-residential counterparts (TinHan et al., 2018). Examination of humpback whale (*Megaptera novaeangliae*) skin samples as well as satellite telemetry throughout the Chesapeake Bay, showed that whales utilize the area for foraging commonly in similar areas to shipping channels, demonstrating related isotopic signatures to whales feeding in the Gulf of St. Lawrence on likely similar prey items (Aschettino et al., 2020). Stable isotopes analyzed in conjunction with movement thus have wide ranging uses across a variety of species with the potential to reveal the ways diet, habitat use, and movement interact.

### Protein Metabolism

Biochemical and metabolic connections with movement have been observed in several fishes, such as in tradeoffs between activity and growth (in yellow perch; Rennie et al., 2005), increased reliance on protein for energy during extended swimming at elevated speeds (in Nile tilapia; Alsop et al., 1999), and protein degradation and synthesis each increased with swimming activity (in rainbow trout; Houlihan and Laurent, 1987). Related to protein metabolism, migration has been linked to increased amino acid and carbon flux in sockeye salmon (reviewed for salmon in Mommsen, 2004). Protein degradation was indirectly linked to walleye (*Sander vitreus*) movement across a large freshwater lake by non-lethal whole blood biopsy (Thorstensen et al., 2021). We speculate that while physiology and biochemistry are certainly affected by and affect movement, specific pathways used to address movement likely differ between species, possibly based on phylogenetic constraints and life history strategies. Protein metabolism has been assessed non-lethally through observations of urea and ammonia excretion in an experimental tank (Alsop et al., 1999), or careful selection of a metabolite panel (Thorstensen et al., 2021). However, research using non-lethal sampling applied to protein metabolism is in its infancy.

### Gene Expression

Internal and external stimuli lead to cellular responses such as changes in gene expression, which reflect the physiological state of an organism (reviewed in Szymański and Barciszewski, 2003; Jeffries et al., 2021). This information on physiological state may be linked to movement in freshwater fishes (see *Physiology and Movement*). Gene expression can be measured by quantifying messenger RNA (mRNA) abundance, which can be sampled from tissues non-lethally (Jeffries et al., 2021). Therefore, techniques that quantify gene activity *via* mRNA expression have great potential for linking movement and physiological state non-lethally in animals.

Quantitative polymerase chain reaction (qPCR), microarrays, and mRNA sequencing are all techniques that have been used to study gene expression and have been paired with movement (Mantione et al., 2014). With these approaches, the abundance of individual genes in groups of fish relative to others are compared, with possible implications for functional response pathways (Miller et al., 2011; Jeffries et al., 2014; Connon et al., 2018). For example, in migrating sockeye salmon, gill gene expression was correlated with environmental changes throughout migration (Evans et al., 2011), and predictive of migration completion (Miller et al., 2011; Jeffries et al., 2014), demonstrating an application of mRNA sequencing for revealing factors influencing migration success. Similarly, hatchery reared steelhead (*O. mykiss*) migration survival was predicted *via* elevated transcript abundance of inflammatory responsive genes in the gill, *via* qPCR, associating gene expression responses with mortality or successful migration (Healy et al., 2018). The combination of these technologies and transcriptional markers for environmental stress, immunity, and pathogen presence provides predictive power, and enable risk assessments of predation throughout migration. These approaches further link molecular signatures of disease status and stress to interspecific predator-prey interactions, especially in wild salmonids (Jeffries et al., 2014; Miller et al., 2014, 2017; Furey et al., 2021). In the Japanese eel (*Anguilla japonica*), upregulation in prolactin gene expression revealed its importance during upstream migration from sea water to fresh water, and its role in relation to hyper-osmoregulation (Yada et al., 2019). Alternative migratory tactics in brown trout (*Salmo trutta*) were associated with gene expression in liver and brain tissue (Wynne et al., 2021). Additionally, alteration of metabolic and immune processes in the liver were identified in genes differentially expressed between migrants and residents of the same species, indicating physiological influences on movement strategies (ibid).

### Integrating Gene Expression and Physiological Indices

Gene expression and movement data have often been integrated with other physiological indices. Migration success, infectious agents, and plasma variables (e.g., cortisol, lactate, potassium, sodium, leukocrit, among others) were analyzed from the gills and blood of Chinook salmon during upstream migration (*O. tshawytscha*) and provided additional evidence that disease status may be an important predictor of migration success in anadromous fish (Bass et al., 2019). Disease and stress state in the gills of migrating sockeye salmon were also investigated with gene expression (*via* microarray and qPCR) and blood plasma parameters paired with telemetry (Drenner et al., 2018). This study provided further evidence for the importance of those parameters in migration and revealed that stressed fish may enter fresh water earlier, but experience subsequently higher mortality (ibid).

Body tissues are also useful in linking gene expression with migration and overall movement. Muscle tissue can aid in linking gene expression with migration success and environmental xenobiotics. This relationship was found in sockeye and Chinook salmon including xenobiotic compounds such as organochlorine pesticides, organohalogen residues, and polybrominated diethyl ethers (Veldhoen et al., 2010). Here, sockeye salmon were found to experience greater biological stress than the Chinook salmon, in conjunction with endocrine disrupting chemicals (ibid). Brain, gill, muscle, and liver tissue from Coho salmon (*O. kisutch*) revealed how gene expression may change with important life history events including implications for physiological preparations for migration (Houde et al., 2019). Other work has also connected physiological and transcriptomic patterns, such as staggered increases in protein turnover, protein biosynthesis, immune responses, oxidative phosphorylation, and glycolytic potential as sockeye salmon underwent anadromous migrations (Miller et al., 2009). These gene expression and physiology-based studies may show a bias towards fish with anadromous migrations, but nevertheless demonstrate the enormous potential for describing how movement may be impacted by different environmental factors during fishes’ lifetimes.

## Methods for Connecting Movement and Genetics

### Population Genetics

Population genetics has been useful for describing the extent to which evolution and movement interact by measuring multigenerational population connectivity (reviewed in Ovenden et al., 2015; Cayuela et al., 2018). Here, connectivity is measured as dispersal, which is often linked to gene flow and population structure (Lowe and Allendorf, 2010; Hall and Beissinger, 2017; Cayuela et al., 2018). With population genetics, the unit of observation is genetic material ‘moving’ between places and populations over generations, while in other studies in this review, the unit of movement is an organism moving within its lifetime. Effective dispersal refers to an individual’s success at reproducing, whereas non-effective dispersal is characterized by a failure to reproduce (Lowe and Allendorf, 2010; Cayuela et al., 2018). Within effective dispersal, historical and recent gene flow are distinguishing characteristics of population connectivity (ibid). Methods for assessing dispersal using population genetics include indirect approaches that assess effective dispersal through its outcome on genotype frequencies and direct approaches that measure all dispersal by assigning individuals to their population of origin (Broquet and Petit, 2009). For example, in yellowfin tuna (*Thunnus albacares*), a direct approach of genetic assignment with DNA data detected asymmetric dispersal between two current stock designations, which had important implications to the management as it suggested spawning stock biomass was overestimated (Mullins et al., 2018). DNA from fin clips has been used to identify how population origin can affect migration behaviours in wild and stocked Atlantic salmon in Europe (Jepsen, 2004) and in brook trout (D’Amelio et al., 2008). Furthermore, by coupling population genetic methods with other approaches such as telemetry or mark-recapture, DNA data can be further used to describe and model species movement (Cayuela et al., 2018; DeSaix et al., 2019). For example, to describe movement in lemon sharks (*Negaprion brevirostris*), Kessel et al. (2014) combined population structure data with acoustic telemetry to develop a residency model for predicting aggregations.

### Quantitative Genetics and Association Tests

Quantitative genetics with experimentally-manipulated pedigrees in Atlantic salmon have revealed a 50–70% heritability for migrant probability (Debes et al., 2020). Variance in migratory traits has been linked with genomics using quantitative genetic approaches in arthropods, fish, and birds, although connections with movement were often indirect (Liedvogel et al., 2011). Association testing in migration timing of Pacific salmon (*Oncorhynchus* spp.) has revealed a strong genomic basis for early versus late-run fish linked with the genes *greb1* and *rock1* (O’Malley et al., 2013; Brieuc et al., 2015; Hess et al., 2016; Prince et al., 2017; Koch and Narum, 2020; Thompson et al., 2020). A chromosomal rearrangement was associated with migration in Atlantic cod (Kess et al., 2019), showing that an association-based approach can also reveal structural genomic variants possibly underlying migration phenotypes. The age at which Atlantic salmon return from marine environments to spawn was associated with muscle development and metabolic genes, among others, and has implications for connections between life history strategies, movement, and genomics (Johnston et al., 2014). Several non-lethal association tests used in birds are presented here, for their possible relevance to conducting future research on freshwater fishes. In two warblers (*Vermivora chrysoptera* & *V. cyanoptera*), a selection-based approach was used to identify the gene *vps13a* as affecting wintering location choices (Toews et al., 2019). A hybrid zone of Swainson’s thrush (*Catharus ustulatus*) was used to identify genomic regions underlying migratory orientation, such as in endopeptidase inhibitor activity and circadian clock genes (Delmore et al., 2016). These different lines of evidence point to a genomic influence on movement in certain circumstances, with implications for potential impacts of genetic variation on the constraint or promotion of movement-based responses to changing environments. Associations between movement and genomics are in their infancy, although some consistent findings may be emerging (Harringmeyer et al., 2021). Certain genomic regions such as those containing GREB1L or circadian rhythm-related genes may be more susceptible to mutations or have an influence on migration (ibid). With more research, these genomic regions may be useful for identifying conservation units for conserving specific adaptive variation in freshwater fishes (Prince et al., 2017).

### Further Considerations

#### Handling Fish

An important property of physiological samples is that they are a ‘snapshot’ of the fish’s physiological status at that time, however acute markers can be sensitive to handling stress during tagging/manipulations (Lower et al., 2005; Stoot et al., 2014; Dick et al., 2018; Lawrence et al., 2018), and are sometimes limited to larger fishes where samples can be obtained (Lawrence et al., 2020). Biopsies are typically taken when the fish is captured and restrained, and is often conducted in concert with tagging or other sampling events (e.g., Miller et al., 2009; Raby et al., 2012). While such biopsies are typically quick (typically a few minutes to perform; Raby et al., 2012; Henderson et al., 2016), anaesthesia can be used for longer sampling workups (Bøe et al., 2020). Importantly, the process of capture, handling, and sampling can impart stress on the animal which may affect post-release mortality and predation (Raby et al., 2014; Hoyle et al., 2015), post-release behaviours (Hoolihan et al., 2011), and alter the physiological status of the sample itself (Dick et al., 2018; Lawrence et al., 2018). Therefore, sampling should always be conducted expediently and in a manner that minimizes harm and stress to the fish itself. Proper sampling techniques and storage of tissue are important considerations that should be incorporated into study designs to ensure that sample parameters are reflective of the treatment of interest and not a methodological artefact (Clark et al., 2011; Schwieterman et al., 2019; Lawrence et al., 2020).

### Collecting Molecular Data

Molecular experiments require that tissue sampling be conducted in a standardized manner that ensures sample quality is maintained. In most cases, ecologically-based studies in the context of this review generally employ non-lethal biopsies such that movement patterns can be linked to physiological state or environmental conditions (Cooke et al., 2005). Consequently, sampling must ensure that enough tissue is collected to fulfil analytical requirements while also minimizing adverse impacts on the animal (Corthals et al., 2015; Lawrence et al., 2020; Jeffries et al., 2021). As some of these analyses (i.e., gene expression) can be quite sensitive to stress or handling effects over even brief stressor durations (Krasnov et al., 2005; Milla et al., 2010; Vehniäinen et al., 2019), it is also important to ensure that sampling is done in an environment which minimizes these effects and allows for a timely collection of tissues (See tagging considerations). Granted, sampling of tissues in ecological studies are unlikely to occur in a completely stress-free environment (e.g., fish caught straight from a river and biopsied on-shore), which makes standardisation of the sampling protocol a key requisite in ensuring that handling-associated impacts remain uniform across all replicates in the study. As for the removal of the tissue biopsy, we recommend that gloves be worn at all times and that sampling tools (i.e., scissors, scalpels, punches, etc.) and frequently handled surfaces be sterilized in 70–95% ethanol in between each sampling event (e.g., Andruszkiewicz et al., 2017; Turner and Bucking, 2019). More specialized cleaners may also be employed such as RNase Away™ (Molecular BioProducts Inc. San Diego, California, United States) in RNA-based experiments, which neutralizes RNases from contacted surfaces (Peterson and Freeman, 2009; Carpenter et al., 2014; Andruszkiewicz et al., 2017) thereby reducing the likelihood of degraded RNA samples. Proper sterilization ensures that residual RNA/DNA/bacteria/degradation enzymes are removed from the surface and reduces the risk of sample contamination. Once removed, sampled tissues need to be quickly stored, which commonly includes either the use of a preserving agent (e.g., RNA*later*; Invitrogen™, Carlsbad, California, United States) or by snap-freezing the tissue with liquid nitrogen. The latter method is thought to be a better means of sample preservation but may not be possible in all field applications because of transportation or safety concerns (Corthals et al., 2015; Passow et al., 2019). See also a study of sample handling best practices to preserve RNA integrity in Vehniäinen et al. (2019). Due to its ease of use and the ability to store samples in the field, RNA*later* is often used in RNA and DNA-based field studies in fish biology (Garseth et al., 2013; Teffer and Miller, 2019; Vehniäinen et al., 2019; Kazyak et al., 2021). By contrast, preserving tissues for DNA sampling can be done with a variety of field-ready methods due to its stability (reviewed in Nagy, 2010). Tissue preservation for DNA extraction is commonly done with 95% ethanol, but freezing, desiccation, and even preservation in vodka or toilet paper are possible, as well (Oakenfull, 1994; Nagy, 2010; Baptista and Goodwin, 2017).

### Ethics and Non-Lethal Sampling

Removal of a large number of fish for research purposes can have unintended consequences, especially in small rivers or lakes (Use of Fishes in Research Committee, 2014). For example, removal of many fish at one trophic level can lead to changes in species abundance at other trophic levels and can even lead to trophic collapse in severe situations in sensitive ecosystems (Dobson et al., 2006; Henderson et al., 2016). In addition, a practical benefit of non-lethal sampling is that it enables data collection after an organism is handled *via* data loggers or tags. In laboratory holding studies, non-lethal sampling allows for monitoring growth and survival post-sampling. For movement studies, this post-handling information is generally where movement data is derived. Other methods, such as isotope-based analyses, only provide information about the organism prior to sampling. Therefore, non-lethal sampling may be both favourable and required for conducting research in practice depending on the question being addressed (Use of Fishes in Research Committee, 2014).

However, a discussion of the ethical considerations underlying non-lethal sampling is useful for considering *why* non-lethal sampling may be important, and in what contexts it may be used. *The Principles of Humane Experimental Technique* (Russell and Burch, 1959) is a widely influential application of ethics to animal research, and guides policy to the present day (Tannenbaum and Bennett, 2015). It describes principles of “reduction”, “refinement”, and “replacement” (the three R’s) for humane animal use in research. In this framework, non-lethal sampling may be considered in the context of refinement, but non-lethal sampling may still cause harm to an animal. The fact the animal survives may actually increase distress by introducing negative mental states (e.g., pain, fear, or isolation). While the question of whether fish can feel pain is discussed widely (Rose et al., 2014; Chatigny, 2019), evidence that tagging and sampling affect post-release behaviours (e.g., Wilson et al., 2017) shows that non-lethal sampling does affect sampled individuals. However, underlying the principles of the three R’s is the idea of wellbeing. Definitions of wellbeing range from an absence of distress to the presence of positive mental states and experiences (Tannenbaum and Bennett, 2015). If wellbeing was interpreted as an absence of distress, then non-lethal sampling can contribute to distress and would not be consistent with wellbeing. However, if wellbeing was considered as positive experiences or mental states (Tannenbaum and Bennett, 2015), non-lethal sampling contributes to wellbeing by allowing future positive experiences for a research organism. While the principles of the three R’s of humane experimental technique are useful heuristics for many research approaches, they are sometimes insufficient for considering why non-lethal sampling is important.

Another idea relevant to non-lethal sampling is intrinsic value, which is the value of organisms beyond what they can do, acknowledging a value in and of themselves (Vucetich et al., 2015). While rarely discussed explicitly, intrinsic value is widely accepted among surveyed people and underlies many wildlife-oriented policies (Vucetich et al., 2015). Intrinsic value is significant because it explains why non-lethal sampling is more ethical than lethal sampling as non-lethal sampling respects the value inherent to individual research organisms. The value of a wild research organism’s life may be considered under other value systems, such as the ecosystem services and contributions to biodiversity it provides upon release (i.e., use value; De Groot et al., 2002; Vucetich et al., 2015). However, intrinsic value is necessary and sufficient to justify non-lethal sampling in organisms regardless of potential ecosystem services. Therefore, intrinsic value may be used along with the principles of humane experimental technique for designing ethical research projects involving wildlife.

### Integrating Data

Integrating datasets is of broad interest to biologists who seek to link movement with other types of information (Figure 1). However, integration is often easier said than done. One potentially useful framework to consider integration is in distinguishing integrative explanations, methods, or data, although other points of view exist (reviewed in O’Malley and Soyer, 2012; Brigandt, 2013). Integrative explanations may be the gold standard for new knowledge but are possibly the most philosophically difficult kinds of integration to achieve. Issues with ultimate and proximate causation, scales of data collected, and diverging perspectives of collaborating researchers are all challenges for scholars that seek integrative explanations (Mayr, 1961; O’Malley and Soyer, 2012). Challenges with data and methods are addressed below, but challenges of diverging perspective and ultimate or proximate causation are project- and domain-specific.

Integration of methods involves either sequential or simultaneous application of different methods on the same biological system (O’Malley and Soyer, 2012). This form of integration is often motivated in part by the techniques available to researchers, as issues of scale and data availability may preclude direct connections between data. Integration of statistical methods can then provide a way to find integrative explanations. For example, in Thorstensen et al. (2021), different scales of time precluded direct inclusion of length-at-age, length-mass relationship, and metabolite concentrations in the same models of walleye in a large lake. Instead, different models were developed within each scale of time, yielding results that showed consistent spatial patterns across the different scales of time with indirect connections to movement (ibid). A similar conceptual approach was used in modeling walleye movements in relation to temperature (Raby et al., 2018). Separate models of outmigration timing from the western basin of a large lake, the extent of eastward migration, and of summer temperatures in the fish’s habitat did not individually integrate all datasets analyzed in the study. But together, the analyses provided an integrative explanation for walleye post-spawning emigration from the western basin as partially driven by temperature (ibid). Direct integration of data with specialized statistical tests, such as in genotype-environment associations (Forester et al., 2018) or in genome-wide associations (Choi et al., 2020) is sometimes possible. Gene expression data can be modeled in an integrative way as well, such as by using weighted gene correlation network analysis to relate correlated modules of genes with external sample traits (e.g., mass, length, experimental treatment) in RNA-seq data (Stuart et al., 2003; Langfelder and Horvath, 2008). These methods have been developed for integrating specific kinds of data and provide appropriate tools to address statistical issues. However, specific methods are not available for many of the possible connections between datasets in biology, and developing these methods is complex and requires deep statistical knowledge. One common approach is to apply multiple statistical tests on a single dataset to find overlapping patterns of significance as a way to integrate methods. In doing so, however, overall significance is limited to the weakest statistical method used (Forester et al., 2018). Nevertheless, the success of integrative methods for specific kinds of data, and the success of integrative explanations from analyzing different statistical methods concurrently, shows that this approach is challenging but rewarding.

Integration of data is the foundational step toward achieving integrative methods and explanations. Here, data must be standardized and made available beyond the original researchers that collected it such as by deposition in repositories (O’Malley and Soyer, 2012). Data standardization is an overlooked challenge as data structures may differ substantially between scientific fields or types of data (Wickham, 2014). As an illustration of challenges in integrating data, observations of temperature at different sites along a river may be recorded as three estimates per site per researcher visit, while observations of movement from an acoustic receiver array may range from data at 10-minute intervals for some fish staying near receivers to weekly observations for fish who happen to use space primarily outside the receiver array. Meaningfully combining these observations across space and time would be a non-trivial data science task, and integrative research often presents data challenges of similar scope. We recommend three basic practices in data formatting to facilitate integration: each variable forms a column, each observation forms a row, and each observational unit forms a table (i.e., tidy data; Wickham, 2014). In the above example for temperature and movement data, one table would contain information on temperature, and another would contain information on fish movement *via* acoustic telemetry. Data repositories can hold overlooked complexity, as well. Out of 1,379 repositories for research data, 832 were discipline-specific (Kindling et al., 2017). Repositories themselves are faced with different issues such as data formatting, documentation, validation, and accessibility (Assante et al., 2016). As such, collating data from across repositories or making data available to a community of researchers within a repository may be an unexpected challenge in integrative research.

### Management

Given the enormous importance of movement to animal survival and reproduction (Nathan et al., 2008), understanding when, where, and why fish move is important to effective long-term management of fisheries resources (Cooke et al., 2016). Collecting and analyzing movement data is a large task though, one that has been addressed by large collaborations such as the Ocean Tracking Network and Great Lakes Acoustic Telemetry Observation System (Cooke et al., 2011; Krueger et al., 2018). With managers involved throughout the research process, relevancy of movement analyses can be maintained (Krueger et al., 2018). Manager participation can include direct involvement in research (e.g., co-authorship), face-to-face communication (e.g., annual meetings), and early participation in researcher networks at the start of the collaboration (ibid). The integrative research reviewed here also has potential applications for management, such as in using physiological thresholds in conservation policy by identifying transcriptomic responses in the context of habitat requirements and movement ecology (Connon et al., 2018). In addition, the genomics of premature migration can be used to potentially define conservation units as a supplement to management frameworks that conserve adaptive variation that maintains the potential for different movement strategies in conserved populations (Prince et al., 2017). As effective management has been guided by the individual components of movement, physiology, and genetics described in this review, research that integrates those approaches has great potential to address future management needs (Figure 1).

Specific examples of movement-based research being applied to management questions exist, as well. Dams and water diversion structures provide important goods and services to humans, but can alter freshwater systems and affect species movement (Grill et al., 2019). River regulation can change flow regimes by altering timing, duration, and frequency of high or low flow conditions, alter water quality, dilute chemical cues, change water temperature, and can even completely impede migration (Hatry et al., 2013; Bennett and Kozak, 2016; Enders et al., 2019; Bett et al., 2020). Not only can non-lethal sampling provide valuable information to inform management decisions while reducing the effects of the research on population size or dynamics, but integration of non-lethal sampling techniques can lead to important management decisions. For example in a long-term study by Bett et al. (2020) monitoring migrating Pacific salmon (*Oncorhynchus* spp.) in Seton River (a tributary of the Fraser River, British Columbia, Canada), researchers integrated multiple techniques of telemetry, behavioural tests, molecular analyses, and hydraulic monitoring to characterize the effects of a dam fishway on the survival and reproductive success of returning adults. Their findings directly influenced new operational guidelines for dam flow releases (ibid), demonstrating that non-lethal sampling with movement information can be a powerful management tool.

## Discussion

Freshwater systems are faced with some of the most pressing contemporary conservation issues and are important for numerous human and ecosystem-based uses. At the landscape scale, these freshwater ecosystems are characterized by waterbodies of varying connectivity, together creating mosaics of fresh water. Movement research on freshwater systems has the potential to explain key factors underlying organism movements within and between waterbodies in these mosaics of connectivity. Non-lethal sampling has enormous potential for addressing integrative movement-oriented questions. Tagging methods require an initial interaction with a research organism before release, a situation consistent with non-lethal practice. During these brief interactions, an opportunity exists for researchers to non-lethally sample a small amount of biological tissue useful for analysis in diverse approaches across environmental toxicology, stress physiology, gene expression, and genetics. Preserving a research organism’s life is practical for species of conservation concern, such as those in small populations that may be diminished by large lethal sample sizes, and ethical within frameworks of wellbeing (which underlies the three R’s) and intrinsic value. Using movement information in conjunction with other data requires careful domain-dependent interpretations of results, and careful but creative applications of integrative methods. Given the importance of movement in freshwater, the conservation issues facing freshwater systems, and the practical and ethical advantages of non-lethal sampling, resource managers would benefit from using non-lethal sampling and movement-centered questions to guide decisions. With the array of biological sampling and tracking methods becoming available to researchers, integrative non-lethal movement studies will provide deep insights into freshwater ecosystems in the future.
